# Quantification of seed–soil contact of sugar beet (*Beta vulgaris*) using X-ray Computed Tomography

**DOI:** 10.1186/s13007-017-0220-4

**Published:** 2017-08-30

**Authors:** Sebastian Blunk, Ali Hafeez Malik, Martine I. de Heer, Tobias Ekblad, Jennifer Bussell, Debbie Sparkes, Kenneth Fredlund, Craig J. Sturrock, Sacha J. Mooney

**Affiliations:** 1 Division of Agriculture and Environmental Science, School of Biosciences, The University of Nottingham, Sutton Bonington, Loughborough, LE12 6HW UK; 2 Syngenta Seeds AB, Sabyholmsvägen 24, Box 302, 261 23 Landskrona, Sweden; 3 Syngenta Crop Protection, Bracknell, RG42 6EY UK

**Keywords:** Sugar beet, Germination, Seeds, Seed–soil contact, X-ray Computed Tomography

## Abstract

**Background:**

Seed–soil contact is important to ensure successful germination, however, there is a paucity of reported studies that have quantified the microstructure at and around this critical interface, mainly due to the opacity of soil.

**Results:**

Here we describe a novel methodology to non-destructively calculate the seed–soil contact area using X-ray Computed Tomography. Under controlled conditions, we observed that seed–soil contact was strongly influenced by the size and type of seed, with a seed–soil contact of ca. 15% for naked sugar beet seeds compared to ca. 32% for pelleted and coated seeds. Similar results were obtained for seeds sampled from the field albeit with a higher spatial variability.

**Conclusions:**

By application of this new quantification method it is hoped seed enhancement technologies can be optimised and ultimately seedbed preparation improved to ensure better germination.

## Introduction

Seeds require specific soil environmental conditions (such as moisture, temperature, pH) to successfully initiate germination and establish into a mature plant. Water absorption (imbibition) is the fundamental first step in this process. During imbibition, the dry seed hydrates and swells resulting in an increase in volume. Due to the absorption process of water from the soil, high levels of seed–soil contact have been reported as a necessary requirement [1, 2]. The choice of cultivation practice, e.g. plough and press versus minimum tillage cultivation practices, are reported to have strong influence on seed germination rate [3, 4]. A positive impact of compaction caused by rolling on different crops (e.g. barley, oats and wheat) on their corresponding emergence rate and yield has been reported previously [5]. As rolling increases soil compaction, an increase in seed–soil contact can be expected but it is important to determine to what degree the soil compaction is beneficial and at what point the seed germination rate decreases [6]. Seedbed preparation varies depending on the soil type as well as the crop. Although globally, there is a move towards reduced tillage practices in general, sugar beet seedbeds are prepared differently according to the soil type. Farmers can make use of frost action to loosen the soil via multiple freeze and thaw cycles or also rely on conventional tillage operations including power harrow and spring tine. The crop is then drilled to a stand (precision sowing of each individual seed).

Sugar beet is usually grown in rows 45–50 cm apart, with seeds placed at a predetermined spacing to achieve the desired plant population as sugar beet cannot compensate for gaps of greater than 45 cm within the row [7]. Growers are advised to aim for a final plant population of 80–100,000 plants per hectare [8]. Hence, establishment of crops in 50 cm rows must exceed 70% to avoid yield loss due to incomplete light interception which reinforces the importance of predictable and uniform establishment [7]. To ensure precision sowing, seed shape altering enhancement technologies e.g. pelleting are frequently utilised. Pelleting materials, consisting mainly of wood fibre, are applied to alter the original seed shape into a shape more suitable for the drilling machine. Besides the application of a pellet, a coating is used to facilitate the distribution of pesticides around the seed [9]. Therefore, a better understanding of seed–soil-contact, and how this relates to germination and establishment, is particularly important for the sugar beet crop since it determines rates of water and gas flow through to the seed and hence the developing embryo.

Historically, the measurement of seed–soil contact has been somewhat subjective with the term “adequate contact” defined by Brown et al. [10]. Although the importance of seed–soil contact is well known, previous work has often been limited to measurement estimations, e.g. Collis-George and Hector [11] showed that *Medicago tribuloides* germination rate is increased with a seed–soil–water ratio (seed–soil water contact defined as wetted area in contact with the seed) above field capacity. Furthermore, Collis-George and Melville [12] suggested that seed–soil contact is sufficient as soon as the imbibition rate can be reached by moisture transfer through soil aggregates. Based on this research, several studies focused on optimising seeding tools like seed openers, drills or press wheels to enhance seed–soil contact [13, 14]. Previously the success of sowing approaches has been based on measurements of soil characteristics (e.g. bulk density, porosity, aggregate size and level of compaction) [10]. Though Brown etal. [10] stated that due to the highly heterogeneous nature of soil aggregates, it is not possible to quantify the actual seed–soil contact. They used modelling approaches to examine seed–soil contact by defining the number of contacts and the contact area. This model approach was further developed by Zhou et al. [15] using a discrete element method (DEM). They predicted that the number of active contacts between soil aggregates and the seed varies between 0 and 33 contacts with a contact area of 0–41 mm^2^ depending on the size of soil aggregates as well as the size of the seed. The calculations were conducted using varying seed diameters with no specific model seed. The calibration was undertaken on a range of different soil textures, the seed, and also the sowing method such as application of press wheel and tillage. This work is the only published case to date where an actual number for seed–soil contact is stated besides Brown etal. [10], however, both lack a method to validate the efficacy of the model.

In recent years, X-ray Computed Tomography (X-ray CT) has been shown to be a rapid, non-destructive and non-invasive method to quantify soil structure allowing for 3D visualisation of seedling development [16]. CT technology has advanced to now facilitate high resolution and larger replicated studies due to reduced scan times suited to determine physical soil properties like porosity and pore size distribution and pore connectivity [17–21]. X-ray CT has also been used in plant studies involving root architecture and temporal root development research which illustrate the benefits of non-invasive imaging [22–26].

The advantages of X-ray CT for analysis of seeds has previously been shown analysing the effect of seed priming, pore spaces within seeds and released dust particles [27–30]. These studies however, have been conducted ex situ, outside soil. Here we propose a new segmentation method using CT images of seeds growing in soil to enable an in situ measurement of contact area based on a similar method by Schmidt et al. [31] for calculating plant root–soil contact. Here we develop this approach to enable the calculation of the precise seed–soil contact for the first time on an untreated naked sugar beet seed as well as a commercial pelleted and coated seed. Finally, the method was evaluated on soil samples taken directly from a recently prepared seedbed in the field.

## Materials and methods

Soil was collected from the top layer of an arable field at the University of Nottingham farm site at Bunny, Nottinghamshire, UK (52.8586°–1.1280°). The soil type was a sandy loam soil of the Newport series (FAO Eutric Cambisol) (78.7% sand, 9.4% silt, 11.9% clay and 2.3% organic matter) [32]. The soil was air-dried and sieved to <1 mm. Sugar beet (*Beta vulgaris*) seeds were supplied by Syngenta Seeds AB, Sweden. Untreated (naked) seeds were compared with pelleted and coated seeds, the latter being the standard product form available on the market (coating and pelleting compositions are treated confidentially). Untreated seeds were star-shaped with a size of approximately 4 mm × 3 mm × 3 mm whereas pelleted and primed seeds were round with a diameter of 4 mm. All seed treatments used in this study are available for order from Syngenta Seeds AB, Sweden, by referring to this study. Polypropylene cylinders were cut to 25 mm diameter × 70 mm length. To allow cylinders to be opened without disturbing the soil within, each cylinder was cut in half longitudinally and the two halves taped together on one side. A detachable mesh was attached using adhesive tape onto a strip of plastic that was folded around the base of the cylinder. The mesh enabled free drainage of water from the base of the cylinder. The cylinders were filled with the sieved sandy loam soil (22.8 g) to a height of 50 mm. A seed was placed centrally on top of the soil and then covered with an additional quantity of soil (6.8 g) resulting in a bulk density of 1.2 Mg m^−3^ for a total equivalent height of 65 mm. The soil was saturated from the bottom of the column for 5 min and then drained to 20% gravimetric moisture content by placing the column on tissue paper determined by weight. The soil columns were placed in a growth room with a day temperature of 20 °C, a night temperature of 15 °C and 16 h of daylight (1 h each for dusk and dawn) for 4 days. The moisture content was monitored daily and maintained at 20% w/w. Additionally, three undisturbed field cores (5 × 5 cm) were extracted by sampling directly behind the tractor mounted drilling machine and used for comparison to the artificially created cores. The sowed seeds were pelleted and coated.

A Phoenix v|tome|x m 240 kV X-ray CT system (GE Measurement & Control Solutions, Wunstorf, Germany) was used to scan each column using a potential energy of 130 kV, a current of 100 µA collecting 2878 projection images over a 360° rotation with a detector timing of 250 ms. Projection image averaging and skip were set to 1 and 0, respectively. The columns were scanned at 20 µm resolution with an acquisition time of 12 min in a multi scan mode for two scans (A scan of the bottom section and a scan of the top section that are combined into one volume to enable a higher spatial resolution than scanning the whole column within a single scan with a larger field of view).

## Results

### Method development

**Fig. 1:**
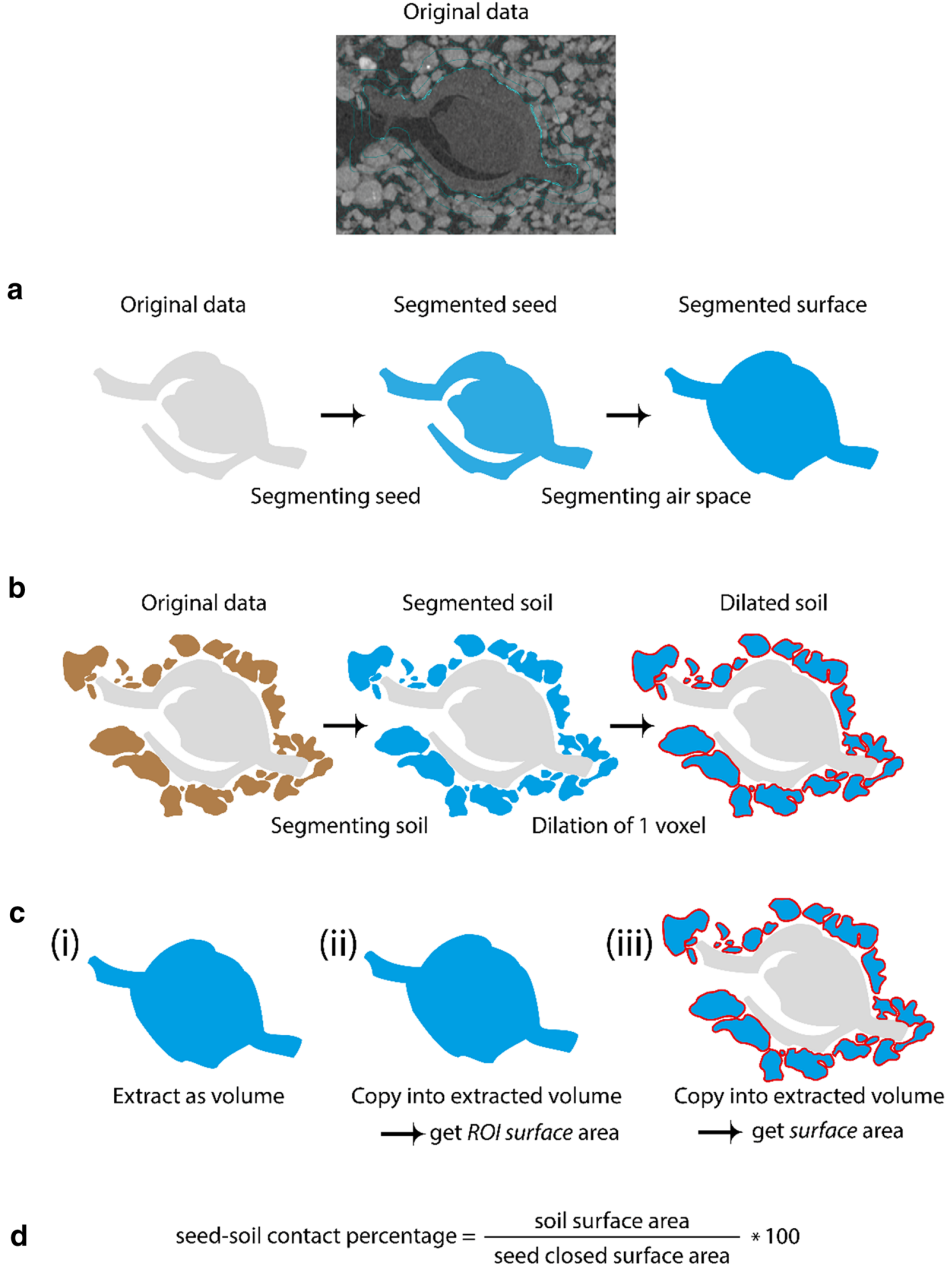
Schematic representation of the seed–soil contact calculation process. The original image shows contact areas in *blue* based on the overlap of dilated soil aggregates. **a** During the process of germination, seeds open to enable radicle penetration which is indicated with the schematic representation of the 2D slice. The standard process of segmentation was conducted with additional segmentation of the air space within the opening seed. The segmented surface was dilated by +1 voxel. **b** *Brown* objects indicate a simplified version of soil aggregates around the seed. Using surface determination, the soil aggregates were segmented and finally dilated by +1 voxel. **c** The dilated surface from step A was extracted as a volume and the segmented surface (3rd step of A) copied into the volume as a ROI. The surface area of the seed is now listed in determine properties as closed surface area. The dilated soil from step B is also copied into the new volume and the surface area determined via determine properties. **d** The previously determined surface properties (step C) were used to calculate a seed–soil contact percentage

Schmidt et al. [31] presented a method to determine the contact area of soil touching the root of a lupin plant. A fundamental difference between applying the algorithm to a seed system instead of a root system is that the seed, upon initiating germination, starts to develop increased air space regions within the previously closed environment. In contrast, a root is typically represented as a solid shape in an X-ray scan showing no air spaces within, unless aerenchyma form. The segmentation of the seed surface was, therefore, extended across this air space so that the resulting object has a closed surface area relevant for determining soil contact (Fig. 1a). The extension was performed using the opening function in *VG StudioMax* ^*®*^ *v2.2* (Volume Graphics GmbH, Heidelberg, Germany) and remaining air space filled manually. In this dataset, the extension after 1 day of growth increased the seed volume by 19.65% (±6.84%) for the untreated seed and 9.07% (±2.10%) for the pelleted and coated seed. The seed surface was thereby decreased by 23.96% (±1.43%) for the untreated seed and 54.92% (±2.10%) for the pelleted and coated seed. The high decrease in seed surface area has no major influence in seed–soil contact as the majority of the filled space is within the seed. The filling of the seed becomes more important throughout the development of the seed as more air space develops over time. Using the surface determination tool in *VG StudioMax* ^*®*^ *v2.2*, the soil was segmented by selecting the air space as background and a selection of different soil aggregates and particles as material. The resulting region of interest (ROI) of the soil was dilated by +1 voxel so that an overlap with the non-dilated seed was created (Fig. 1b). The ROI of the seed surface was then extracted as a volume and both the segmented surface and the dilated soil surface were copied into the new volume (Fig. 1c). By calculating the ratio of the closed surface area of the seed and the surface area of the dilated soil aggregates within the new volume being in contact with the seed surface, a seed–soil contact percentage of the total seed surface was calculated (Fig. 1d).

**Fig. 2:**
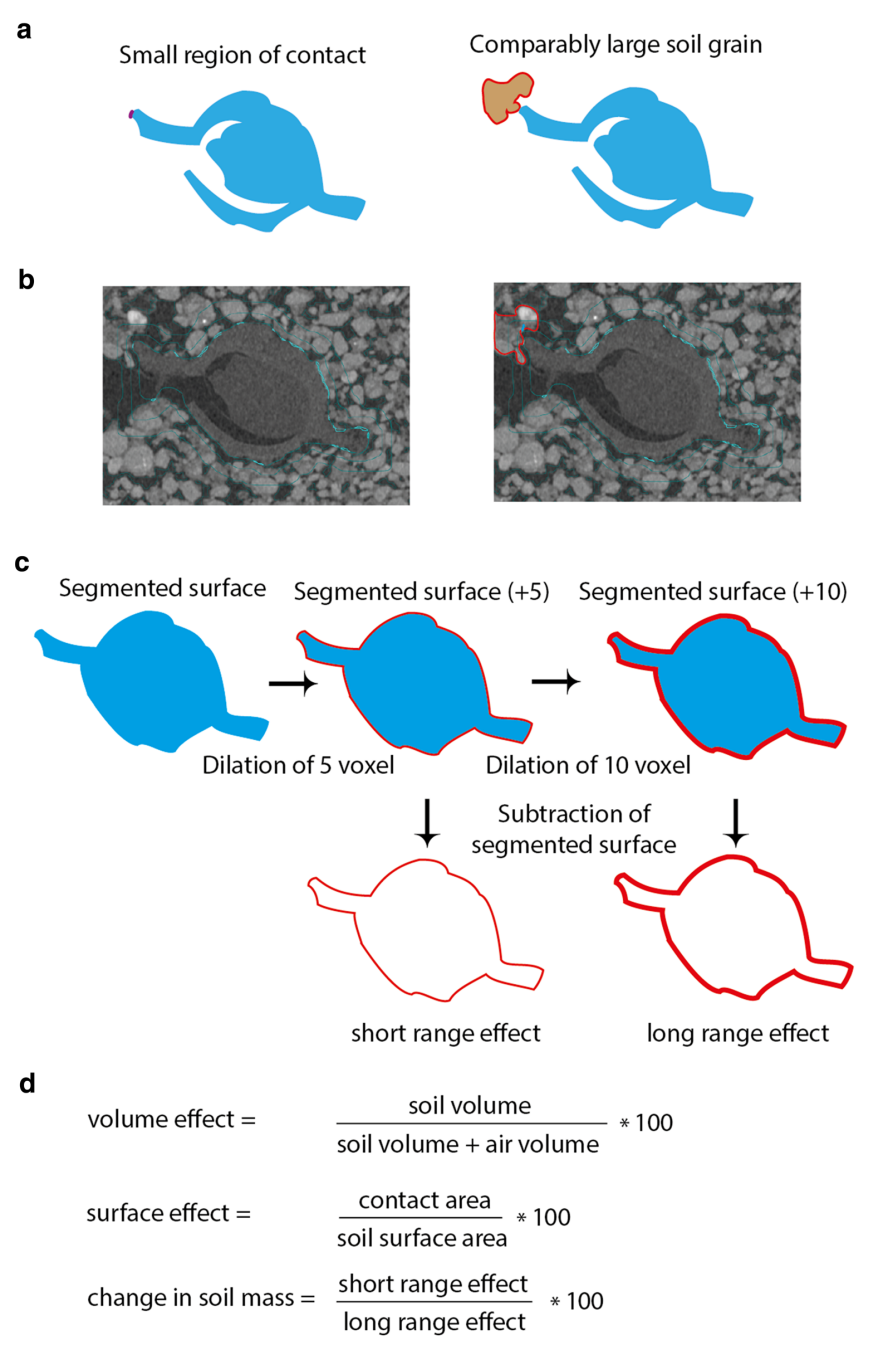
The underlying “iceberg effect”. **a** Schematic representation of the iceberg effect based on 2D slices showing in **b**. **b** Example 2D slice showing contact are in blue and example underlying soil grain behind a single contact point (*red*). **c** Schematic representation of creating rings. **d** Calculations for the volume and surface effect as well as the change in soil mass based on the short range and long range ring created in step C

Real soil aggregates are not uniform in shape as Zhou et al. [15] described in their modelling approach. Therefore, it is likely that only a section of the soil aggregate is in contact with the seed although the complete soil aggregate has a far greater size. This means that the water uptake of the seed through this aggregate as a whole is higher than estimated by considering only the size of the contact area (Fig. 2a). We refer to this as the *iceberg effect* which would result in an underestimation of the water uptake rate if only considering the seed–soil contact percentage (Fig. 2b). To avoid this, we propose that the seed–soil contact should be correlated with the air space–soil space ratio of the surrounding volume as well as a contact surface area—soil space surface area ratio. The X-ray CT data was therefore used to generate an artificial ring surrounding the segmented seed surface to calculate the soil volume and the air volume. Two rings were created spanning 5 voxel (equal to 100 µm) (referred to as ‘short range’) and 15 voxel (equal to 300 µm) (referred to as ‘long range’) representing 10 and 30% diameter of the maximum size of the sieved grains of <1 mm (Fig. 2c). The resulting rings were used for surface determination as described earlier for the segmentation of the soil. The air space was used as a background and several locations of the soil were combined for the surface determination of the soil aggregates. A second ROI was created with a threshold using the minimal and maximal greyscale values from which the soil ROI was subtracted to derive soil and air volume. The volumes were then used to generate the ratio between soil and air space to generate a volume effect estimation of the iceberg effect. In addition, a ratio of the contact surface area divided by the soil surface area within each corresponding ring was calculated. This is referred to as the surface effect of the iceberg effect. The change in soil mass was calculated as the ratio between the short range and the long range effect (Fig. 2d).

### Method application

**Fig. 3:**
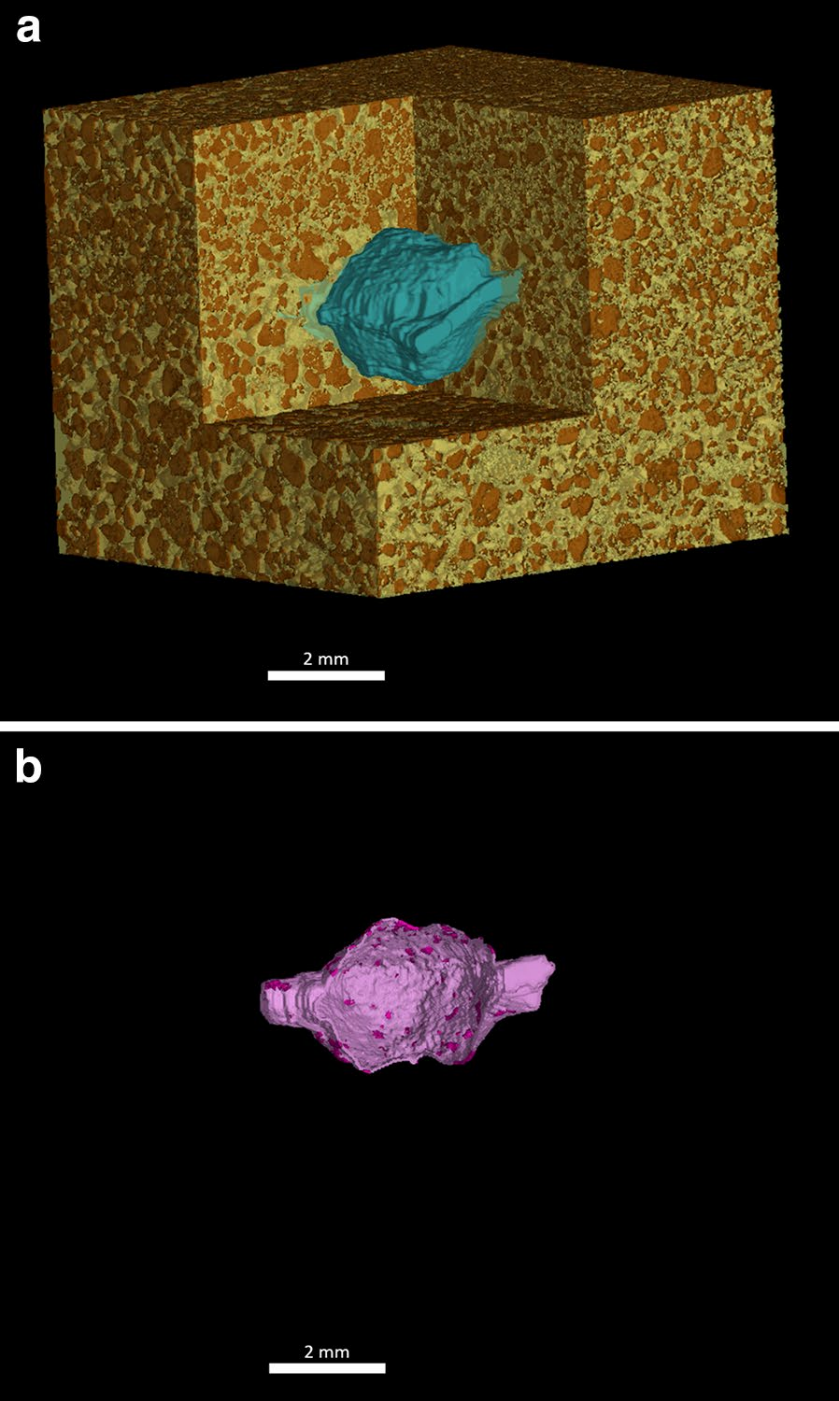
3D visualisation of seed–soil contact. The *images* show an extract of the soil core holding a seedling (naked seed) one day after sowing. **a** Extracted soil core of the column containing the sugar beet seed. *Brown* = Soil, *Yellow* = Air, *Blue* = Seed. **b** Extracted region in contact with the seed. *Pink* = Air in contact with seed, *Purple* = Soil in contact with seed

**Fig. 4:**
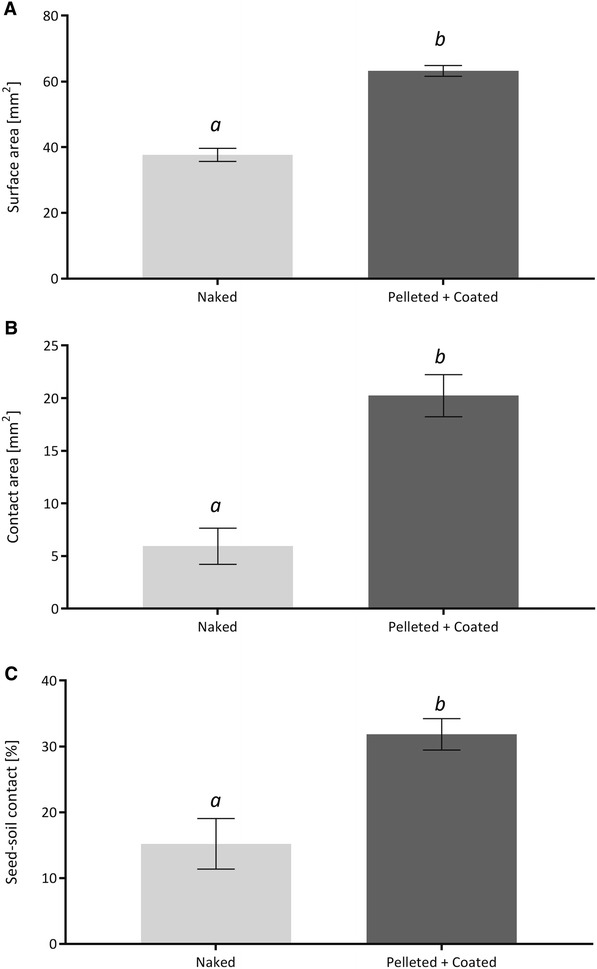
Example contact ratio of naked and pelleted and coated sugar beet seeds. The seeds have grown for 1 day to ensure that the surrounding environment had sufficient time to settle. **A** Measured surface area of the seed. **B** Measured seed–soil contact area. **C** Ratio of contact area divided by surface area as a percentage. *Error bars* calculated for standard error deviation. N = 4

The image processing method was then applied on naked and pelleted and coated sugar beet seeds to visualise and calculate the seed–soil contact (Fig. 3). Figure 4A displays the segmented surface area which showed a significantly higher value for the pelleted and coated seeds compared to the naked seed, 63.22 mm^2^ (±2.00 mm^2^) and 37.67 mm^2^ (±1.63 mm^2^), respectively. The amount of soil which is in direct contact with the seed was significantly higher due to the higher size of the seed (*p* < 0.001) (Fig. 4B). Using both the surface area and the contact area, a contact percentage was calculated showing pelleted and coated seeds had double the contact with the soil than naked seeds (*p* = 0.003) (Fig. 4C). While naked seeds had a contact area of about 15.19% (±3.84%), the pelleted and coated treatment had 31.85% (±2.39%), with both the surface area and the contact area significantly higher (*p* < 0.001) (Fig. 4).

### Iceberg effect calculations

**Fig. 5:**
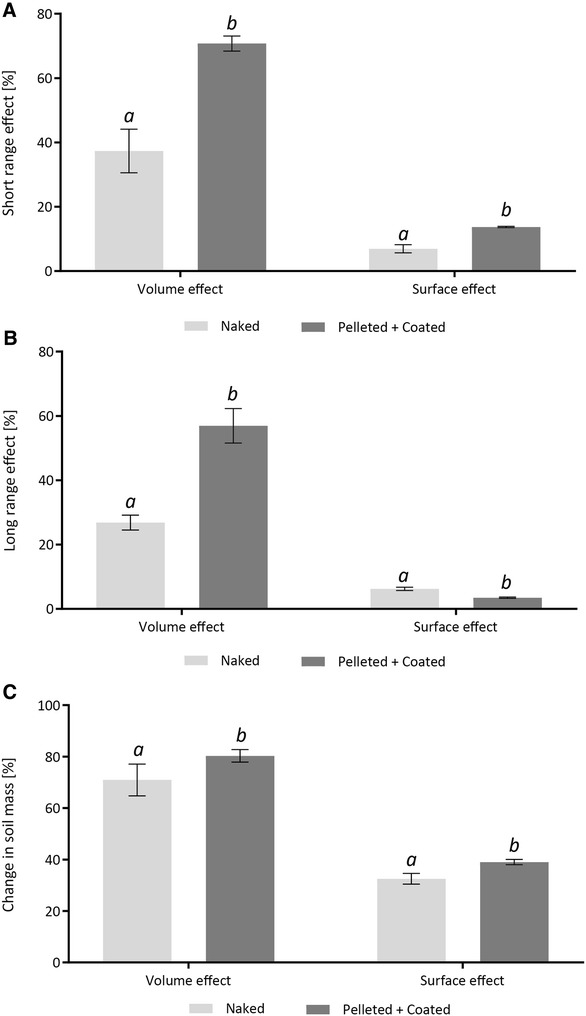
Example iceberg effect calculations of naked and pelleted and coated sugar beet seeds based on contact area, a ring of 100 µm and a ring of 300 µm width. The seeds have grown for 1 day to ensure that the surrounding environment had sufficient time to settle. **A** Contact area divided by soil volume percentage of the 100 µm ring displayed as the volume effect percentage and contact area divided by soil surface within the 100 µm ring displayed as the surface effect percentage. **B** Contact area divided by soil volume percentage of the 300 µm ring displayed as the volume effect percentage and contact area divided by soil surface within the 300 µm ring displayed as the surface effect percentage. **C** Soil volume percentage of the 100 µm ring divided by soil volume percentage of the 300 µm ring as well as surface area of the 100 µm ring divided by the soil surface area of the 300 µm ring. *Error bars* calculated for standard error deviation. N = 4

The ‘iceberg effect’ was calculated for both treatments. The short range effect was calculated by creating the ratio of area of contact, and seed to soil volume ratio in the smaller ring showed a significantly higher percentage for pelleted and coated seeds than for naked seeds, regarding the volume effect (*p* < 0.001) (Fig. 5A). The equivalent long range effect, calculated using the larger ring-shaped region of interest, resulted in a similar significant percentage difference (*p* < 0.001) (Fig. 5B). A similar behaviour was observed for the surface effect for both the short range and the long range effect (*p* < 0.001 in both cases). The change in soil mass between both rings showed a significantly larger percentage for pelleted and coated seeds as well in both effect calculations (*p* < 0.001 in both cases) (Fig. 5C).

### Correlation to radicle growth

**Fig. 6:**
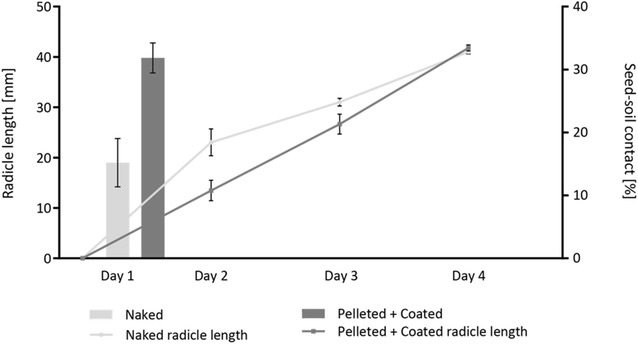
Comparison of the seed–soil contact percentage of day 1 to the radicle length for following days. The radicle length is represented as a *line graph* on the primary axis. The contact percentage is represented as a *bar chart* on the secondary axis. *Error bars* calculated for standard error deviation. N = 4

Figure 6 shows a comparison between the previously calculated seed–soil contact ratio and radicle growth (determined using the polyline tool in *VG StudioMax* ^*®*^ *v2.2*) daily up to 4 days after sowing which demonstrates that even with higher seed–soil contact in pelleted and coated treatments, the radicle length had a slower initial growth. In comparison, the naked treatment with the lower percentage displayed a faster growth rate but resulted in similar final lengths of 41.03 mm (±0.89 mm) and 41.80 mm (±1.20 mm), respectively.

### Comparison to field cores

**Fig. 7:**
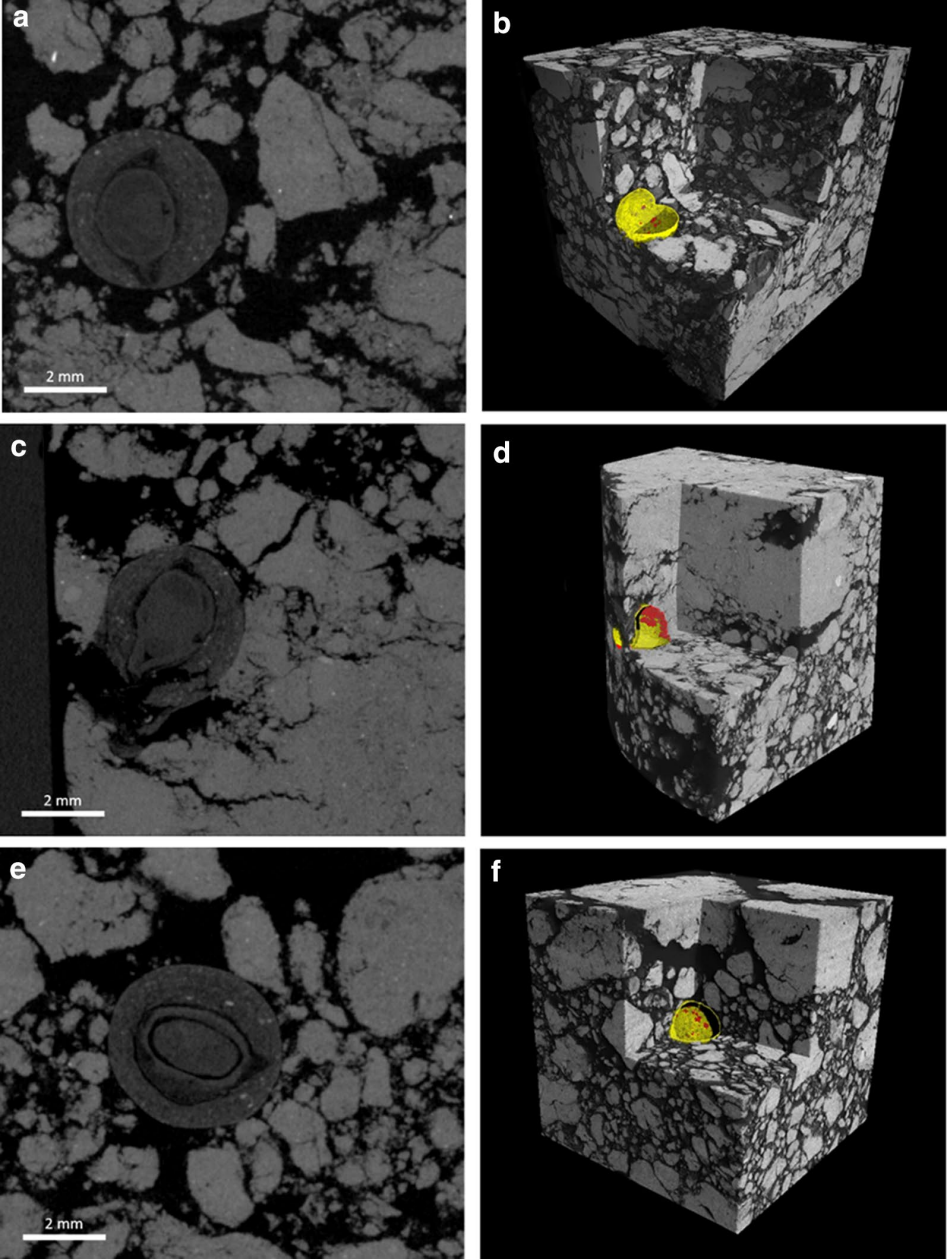
2D and 3D representation of pelleted and coated commercial sugar beet seeds in field cores sampled directly after sowing by drilling. Sampling was executed blindly which resulted in non-uniform positioning of the seeds in the column. **a**, **c** and **e** represent 2D slices of X-ray scans showing different levels of contact. **b**, **d** and **f** display 3D representations indicating soil (*greyscale*), air space (*yellow*), soil in contact with the seed (*red*)

**Fig. 8:**
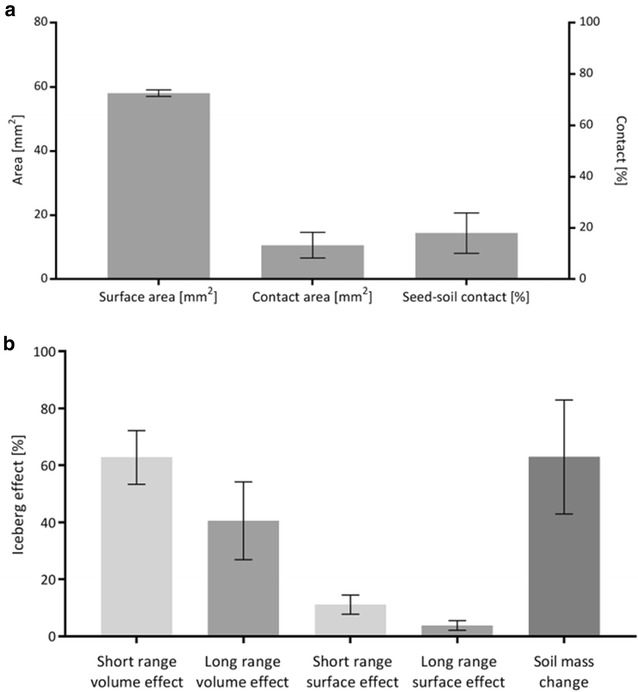
Seed–soil contact calculations based on X-ray scans of field cores with a pelleted and coated sugar beet seed. **a** Showing the seed–soil contact calculations based on the described method showing the surface area, the contact area and the seed–soil contact percentage. **b** Displays the iceberg effect calculations showing the contact area divided by soil volume percentage of the 100 µm ring, the soil volume percentage of the 100 µm ring divided by soil volume percentage of the 300 µm ring and the contact area divided by soil volume percentage of the 300 µm ring displayed as a percentage. *Error bars* calculated for standard error deviation. N = 3

In comparison to the soil columns with sieved (<1 mm) soil, field cores were collected directly from the field immediately after planting and processed using the method described above. As the coring was performed without knowledge of the precise location of the seed in the soil, the positioning of the seed in the core could not be controlled to the same level of precision as with the laboratory prepared cores. Figure 7a, c and e show example 2D slices of the field cores. The seed displayed in Fig. 7c was positioned very close to the edge of the column which could have resulted in soil movement around the seed and therefore alteration of the original seed–soil contact percentage. The seed–soil method was applied to the field cores to calculate a seed–soil contact percentage of 4.79% (Fig. 7a), 31.96% (Fig. 7c) and 17.89% (Fig. 7e). This resulted in an average of 18.21% (±7.84%) (Fig. 8a). The contact within the column is visualised in Fig. 7b, d and f showing that the alteration in soil structure around the seed in Fig. 7d resulted in comparably large areas of contact, hence increasing the seed–soil contact. Calculations of the short range iceberg effect percentages showed slightly lower levels (volume effect: 62.82% (±9.44%); surface effect: 11.15% (±3.38%)) compared to the pelleted and coated seeds in soil columns prepared in the lab (volume effect: 70.75% (±2.36%); surface effect: 13.72% (±0.20%)) as well as for the long range effect (volume effect: 40.59% (±13.64%); surface effect: 3.83% (±1.73%) and volume effect: 56.94% (±6.25%); surface effect: 5.35% (±0.18%), respectively) shown in Fig. 8.

## Discussion

This work suggests that the actual seed–soil contact is very low, challenging previous subjective classifications like “good” and “adequate” seed–soil contact which suggests a higher rate of contact. Brown et al. [10] and Zhou et al. [15] modelling efforts assume that each soil grain has a uniform size and shape. However, as soil is highly heterogeneous most models fail to account for its complexity [33, 34]. X-ray CT can overcome this to an extent and allows the quantification of the actual 3D soil structural geometry. Zhou et al. [15] assumed a contact area of 0–41 mm^2^ which is supported by our measurements of 5.93 mm^2^ (±1.72 mm^2^) for naked seeds and 20.24 mm^2^ (±2.01 mm^2^) for pelleted and coated seeds.

A comparison with field collected cores showed that, although precision sowing and uniform seedbed preparation was executed, a high variability in seed–soil contact for pelleted and coated seeds under field conditions can be observed (acknowledging the low replication number of three here). The variability ranged from 4.79% which is a third of the seed–soil contact in naked seeds to 31.96% which was measured as the average value for pelleted and coated seeds under optimal laboratory conditions. However differences between laboratory conditions using <1 mm sieved soil in comparison to field conditions with formations of macro aggregates with a larger size than the seed are to be expected.

In the literature, seed–soil contact has been previously described as highly influential for successful germination. The low seed–soil contact percentage measured here, however, indicates that that seed–soil contact percentages alone are not sufficient to judge the influence of soil on germination. Therefore, virtual ring calculations were executed showing a correlation between the contact and connected soil aggregates which would enable water transfer towards the seed. The size of the sample ring was determined based on the maximum size of the largest soil aggregate in the column. This however, cannot be guaranteed for field cores as the size of soil aggregates are unable to serve as a reference here due to their large size. In our study 5 and 15 voxels were reasonable distances to identify greyscale value changes where the resolution does not exceed approximately 50 µm. The amount of soil around the seed was higher in the pelleted and coated treatment compared to the naked treatment which we attribute to the larger compressive force exerted on the surrounding soil during the opening of the seed. The iceberg effect calculations suggest that pelleted and coated seeds had access to 70.75% (±2.36%) of the available water and nutrients in the soil whereas the naked seeds only 37.34% (±6.79%) calculated in a short range of 5 voxel regarding the volume effect. The change in soil mass in both treatments is almost similar resulting therefore in a similar access in a longer range of 15 voxel. It was slightly higher for pelleted and coated seeds which was likely to be related to seed opening and therefore the resulting compaction. Hence, the connectivity of the soil aggregates that were in contact with those in immediate distance is higher and therefore the accessibility to a water source was greater in pelleted and coated seeds regarding the absorption through aggregates. Water films are another option for water uptake which cannot at present be taken into account using the iceberg effect calculations as the X-ray CT method is unable to accurately segment water trapped in the small pores within the soil mineral and organic matrix at the resolution employed in this study due to their similar X-ray attenuation characteristics. The water availability here is attributed to the soil volume or soil surface around the seed as soil aggregates hold water by absorbing it. The higher amount of soil volume can be attributed to the large difference in seed–soil contact and therefore the water availability. The short range iceberg effect (5 voxel, 100 µm) in the field cores was 62.82% (±9.44%) which is similar to the artificial pelleted and coated columns. However, due to the lower initial seed–soil contact percentage of 18.21% (±7.84%) in comparison to 31.85% (±2.39%) for the laboratory condition, the seed in the field is likely to have had less access to water. A similar effect could be observed for the surface effect in all calculations. The iceberg effect calculations however, refer also to those soil grains and aggregates that are in close proximity to the seed but not in direct contact so that the iceberg effect is overestimated. These calculations are limited to an extent by the achievable contrast and resolution where pores of a smaller size than the resolution, that maybe holding water, are not considered. Nevertheless, a higher amount of soil due to compaction could be interpreted as a negative trait as air space is also needed to ensure a vapour movement towards the seeds which has previously been proposed as the main source of water in unsaturated soils [35].

A comparison of seed–soil contact on day one after sowing with the radicle length over time showed a slower initial growth speed for the pelleted and coated treatment due to the physical barrier of the pelleting and coating which is applied to enhance the ease of planting as well as the coating for delivery of crop protection chemicals [9, 36]. However, the radicle length of both seedlings of pelleted and coated and naked seeds reached a similar size, which might be due to enhanced water accessibility for the seed. Radicle length observations in this study were limited to 4 days growth and thus are not representative for later growth stages.

The yield of sugar beet is directly related to radiation interception by the canopy [37]. Therefore, the rapid development of a canopy capable of intercepting the majority of incident radiation is essential to maximise yield. Unlike crops such as wheat and oilseed rape, sugar beet does not produce tillers or branches, and hence its ability to compensate for gaps due to poor seedling establishment is limited. Therefore, establishing the optimum plant population of 80–100,000 plants per hectare [8], uniformly across a field, is a fundamental step towards achieving the yield potential of a given crop. Here we have shown our method for quantifying seed–soil contact is applicable to the seedbeds in farmers’ fields. By developing the seed–soil contact method and the understanding of surrounding influences, seedbed preparation methods and farmland management techniques could be adjusted especially regarding the impact of rolling, causing an increase in soil strength. With increased soil strength and increased bulk density, seed–soil contact is meant to increase. However, increased bulk density decreases the oxygen and water supply to the seed therefore increased seed–soil contact could have negative influences. Examining the influence radius for the iceberg effect and the difference of seed–soil contact under different soil conditions would benefit seedbed preparation. Understanding how the properties of a seedbed impact on the seed–soil contact will enable the development of appropriate soil management techniques to create better seedbeds in the future, hopefully facilitating rapid and uniform germination.

## Conclusions

We present a new approach to quantify the in situ seed–soil contact using X-ray CT imagery. We have demonstrated this on sugar beet seeds with different shapes due to seed enhancement technologies however the approach is applicable across a range of plant species. The shape of seeds have an important influence on the actual seed–soil contact, where a round shaped pelleted and coated seed had a higher seed–soil contact area (i.e. area of soil touching the seed) and also a higher contact percentage (percentage of soil touching the seed in relation to the whole surface) than naked star-shaped seeds. This method was also confirmed on field structured soil which exhibited a high variability despite of careful and even seedbed preparation. We introduce the concept of the iceberg effect in the context of seed–soil contact for the first time. The iceberg effect showed a higher soil volume around a pelleted and coated seed than an untreated naked seed which might indicate an increased access to water. Future application of using this technique should be used to support determination of the optimum seed–soil contact for enhanced germination, ultimately enabling farmers to enhance the preparation of the seedbed supporting yield improvement.

## Abbreviations

ROI: region of interest
X-ray CT: X-ray Computed Tomography

## References

[1] Bewley JD, Bradford K, Hilhorst H, Nonogaki H. Seeds—physiology of development, germination and dormancy. 3rd ed. New York: Springer. 2013. Retrieved from http://www.springer.com/us/book/9781461446927.

[2] Smýkal P, Vernoud V, Blair MW, Soukup A, Thompson RD (2014). The role of the testa during development and in establishment of dormancy of the legume seed. Front Plant Sci.

[3] Romaneckas K (2002). Žemės dirbimo optimizavimas cukriniams runkeliams. ŽEMĖS ŪKIO MOKSLAI.

[4] Draycott A (2006). Sugar Beet. Exp Agric.

[5] Hakansson I, Myrbeck A, Etana A (2002). A review of research on seedbed preparation for small grain in Sweden. Soil Tillage Res.

[6] Romaneckas K (2009). Impact of sowing depth and seedbed rolling on sugar beetpdf. Zemdirb Agric.

[7] Jaggard KW. Seed rates and optimal spatial arrangement of seeds for maximum yield and profitability. BBRO 10/04: Review of past studies. 2011.

[8] BBBRO. Sugar beet reference book. British Beet Research Organisation. http://bbro.co.uk/publications/reference-book/. 2016.

[9] Hill HJ (1999). Recent developments in seed technology. J N Seeds.

[10] Brown AD, Dexter AR, Chamen WCT, Spoor G (1996). Effect of soil macroporosity and aggregate size on seed–soil contact. Soil Tillage Res.

[11] Collis-George N, Hector J (1966). Germination of seeds as influenced by matric potential and by area of contact between seed and soil water. Aust J Soil Res.

[12] Collis-George N, Melville M (1975). Water absorption by swelling seeds. I. Constant surface boundary condition. Aust J Soil Res.

[13] Tessier S, Saxton K, Papendick R, Hyde G (1991). Zero-tillage furrow opener effects on seed environment and wheat emergence. Soil Tillage Res.

[14] Doan V, Chen Y, Irvine B (2005). Effect of residue type on the performance of no-till seeder openers. Can Biosyst Eng.

[15] Zhou H, Chen Y, Sadek MA (2014). Modelling of soil–seed contact using the discrete element method (DEM). Biosyst Eng.

[16] Blunk S, Malik AH, de Heer MI, Ekblad T, Fredlund K, Mooney SJ, Sturrock CJ (2017). Quantification of differences in germination behaviour of pelleted and coated sugar beet seeds using X-ray Computed Tomography (X-Ray CT). Biomed Phys Eng Express.

[17] Garbout A, Munkholm LJ, Hansen SB (2013). Temporal dynamics for soil aggregates determined using X-ray CT scanning. Geoderma.

[18] Helliwell JR, Sturrock CJ, Grayling KM, Tracy SR, Flavel RJ, Young IM, Mooney SJ (2013). Applications of X-ray Computed Tomography for examining biophysical interactions and structural development in soil systems: a review. Eur J Soil Sci.

[19] Wildenschild D, Sheppard AP (2013). X-ray imaging and analysis techniques for quantifying pore-scale structure and processes in subsurface porous medium systems. Adv Water Resour.

[20] Felde VJMNL, Peth S, Uteau-Puschmann D, Drahorad S, Felix-Henningsen P (2014). Soil microstructure as an under-explored feature of biological soil crust hydrological properties: case study from the NW Negev Desert. Biodivers Conserv.

[21] Wieland R, Rogasik H (2014). Method for analyzing soil structure according to the size of structural elements. Comput Geosci.

[22] Mooney SJ, Pridmore TP, Helliwell J, Bennett MJ (2012). Developing X-ray Computed Tomography to non-invasively image 3-D root systems architecture in soil. Plant Soil.

[23] Tracy SR, Black CR, Roberts JA, Sturrock C, Mairhofer S, Craigon J, Mooney SJ (2012). Quantifying the impact of soil compaction on root system architecture in tomato (*Solanum lycopersicum*) by X-ray micro-computed tomography. Ann Bot.

[24] Zappala S, Mairhofer S, Tracy S, Sturrock CJ, Bennett M, Pridmore T, Mooney SJ (2013). Quantifying the effect of soil moisture content on segmenting root system architecture in X-ray Computed Tomography images. Plant Soil.

[25] Koebernick N, Weller U, Huber K, Schlüter S, Vogel H-J, Jahn R, Vetterlein D (2014). In situ visualization and quantification of three-dimensional root system architecture and growth using X-ray Computed Tomography. Vadose Zone J.

[26] Pfeifer J, Kirchgessner N, Colombi T, Walter A (2015). Rapid phenotyping of crop root systems in undisturbed field soils using X-ray Computed Tomography. Plant Methods.

[27] Gagliardi B, Marcos-Filho J (2011). Relationship between germination and bell pepper seed structure assessed by the X-ray test. Sci Agric.

[28] Verboven P, Herremans E, Borisjuk L, Helfen L, Ho QT, Tschiersch H, Rolletschek H (2013). Void space inside the developing seed of Brassica napus and the modelling of its function. N Phytologist.

[29] Galhaut L, de Lespinay A, Walker DJ, Bernal MP, Correal E, Lutts S (2014). Seed priming of *Trifolium repens* L. Improved germination and early seedling growth on heavy metal-contaminated soil. Water Air Soil Pollut.

[30] Devarrewaere W, Foqué D, Heimbach U, Cantre D, Nicolai B, Nuyttens D, Verboven P (2015). Quantitative 3D shape description of dust particles from treated seeds by means of X-ray micro-CT. Environ Sci Technol.

[31] Schmidt S, Bengough AG, Gregory PJ, Grinev DV, Otten W (2012). Estimating root–soil contact from 3D X-ray microtomographs. Eur J Soil Sci.

[32] Mooney SJ, Morris C. Quantification of preferential flow in undisturbed soil columns using dye tracers and image analysis. SuperSoil 2004: 3rd Australian New Zealand Soils Conference, 5–9 December 2004 (2008).

[33] Young IM, Crawford JW (2004). Interactions and self-organization in the soil–microbe complex. Science.

[34] Crawford JW, Deacon L, Grinev D, Harris JA, Ritz K, Singh BK, Young I (2012). Microbial diversity affects self-organization of the soil–microbe system with consequences for function. J R Soc Interface.

[35] Wuest S (2007). Vapour is the principal source of water imbibed by seeds in unsaturated soils. Seed Sci Res.

[36] Gorim L. Effects of seed coating on germination and early seedling growth in cereals. Dissertation 2014;1–132.

[37] Scott RK, Jaggard KW, Cooke DA, Scott RK (1993). Crop physiology and agronomy. The sugar beet crop: science into practice.

